# Reducing disease burden in an influenza pandemic by targeted delivery of neuraminidase inhibitors: mathematical models in the Australian context

**DOI:** 10.1186/s12879-016-1866-7

**Published:** 2016-10-10

**Authors:** Robert Moss, James M. McCaw, Allen C. Cheng, Aeron C. Hurt, Jodie McVernon

**Affiliations:** 1 Modelling and Simulation Unit Centre for Epidemiology and Biostatistics Melbourne School of Population and Global Health, The University of Melbourne, Level 3, 207 Bouverie St, Melbourne, 3010 Victoria Australia; 2 School of Mathematics and Statistics, The University of Melbourne, Melbourne, Australia; 3 Murdoch Childrens Research Institute, Melbourne, Australia; 4 Infectious Disease Epidemiology Unit, Department of Epidemiology and Preventive Medicine, Monash University, Melbourne, Australia; 5 Infection Prevention and Healthcare Epidemiology Unit, Alfred Health, Melbourne, Australia; 6 WHO Collaborating Centre for Reference and Research on Influenza, Peter Doherty Institute, Melbourne, Australia

**Keywords:** Influenza, Pandemic preparedness, Neuraminidase inhibitor, Emergency response

## Abstract

**Background:**

Many nations maintain stockpiles of neuraminidase inhibitor (NAI) antiviral agents for use in influenza pandemics to reduce transmission and mitigate the course of clinical infection. Pandemic preparedness plans include the use of these stockpiles to deliver proportionate responses, informed by emerging evidence of clinical impact. Recent uncertainty about the effectiveness of NAIs has prompted these nations to reconsider the role of NAIs in pandemic response, with implications for pandemic planning and for NAI stockpile size.

**Methods:**

We combined a dynamic model of influenza epidemiology with a model of the clinical care pathways in the Australian health care system to identify effective NAI strategies for reducing morbidity and mortality in pandemic events, and the stockpile requirements for these strategies. The models were informed by a 2015 assessment of NAI effectiveness against susceptibility, pathogenicity, and transmission of influenza.

**Results:**

Liberal distribution of NAIs for early treatment in outpatient settings yielded the greatest benefits in all of the considered scenarios. Restriction of community-based treatment to risk groups was effective in those groups, but failed to prevent the large proportion of cases arising from lower risk individuals who comprise the majority of the population.

**Conclusions:**

These targeted strategies are only effective if they can be deployed within the constraints of existing health care infrastructure. This finding highlights the critical importance of identifying optimal models of care delivery for effective emergency health care response.

**Electronic supplementary material:**

The online version of this article (doi:10.1186/s12879-016-1866-7) contains supplementary material, which is available to authorized users.

## Background

Many developed nations maintain stockpiles of neuraminidase inhibitor (NAI) antiviral agents for use in the event of an influenza pandemic, and have developed management plans for using these stockpiles to deliver a *proportionate response*, informed by emerging evidence of likely clinical impact. In light of recent conflicting messages concerning the effectiveness of NAIs for reducing transmission and mitigating the course of clinical infection of pandemic influenza [1–4], many of these nations are now re-evaluating the best use of NAIs in pandemic response, with implications for future stockpile size.

The effectiveness of antiviral interventions on the transmission of pandemic influenza and the resulting burden on health care settings has previously been studied in the Australian context [5–8]. These studies have shown that NAIs are likely to be effective in constraining transmission of a pandemic virus only in a relatively small proportion of low transmissibility, high severity scenarios (where infection is highly visible to the health care system and the basic reproduction number is not much greater than 1). Accordingly, the focus has shifted from using NAIs for containment (e.g., limiting transmission until vaccines become available) to using them to mitigate complications and population impact. In these scenarios, where pharmaceutical interventions are likely unable to reduce transmission, evidence supports some degree of clinical effectiveness against complications and death [9] and indicates that early administration of NAIs to reduce hospitalisations, severe outcomes and death is an appropriate strategy [10]. The benefits of such treatment appear greatest in individuals with underlying risk conditions [10].

Here, we use an updated assessment of parameter estimates of NAI effectiveness against susceptibility, pathogenicity, and transmission of influenza, to inform a dynamic model of influenza epidemiology in combination with a model of clinical-care pathways through the health care system. NAIs are distributed within the consultation and admission constraints of the Australian health care system, as estimated from public health care reports. This model is used to identify effective NAI strategies for reducing morbidity and mortality, and to determine the stockpile requirements (within plausible bounds) to achieve these goals. Our focus is on responding to the *first* pandemic wave, prior to the availability of a definitive vaccine intervention. The outcomes of this analysis are then evaluated in the global context of recommendations for updated pandemic preparedness plans in other high-income countries.

## Methods

In accordance with the Australian Health Management Plan for Pandemic Influenza (AHMPPI) [11], we assume that all identified cases are provided with treatment and post-exposure prophylaxis (PEP) is provided to all identifiable contacts during the first four weeks of the pandemic (the *Initial Action Stage*). During the subsequent *Targeted Action Stage*, treatment and prophylaxis recommendations are rationalised and targeted for maximum effect for the remainder of the pandemic, based on impact assessment and the observed epidemiology.

For the purposes of this study, the Australian population was stratified into five distinct risk groups, whose sizes were informed by June 2014 demographic data from the Australian Bureau of Statistics [12]: Young children aged 0–4 years comprise 6.5 % of the population, for whom NAI treatment is assumed to confer no beneficial effects [10, 13, 14]. Elderly aged 66+ years comprise 13.7 % of the population, for whom NAI treatment is assumed to confer the same benefits as per the general population. High Risk aged 5–65 years comprise 10 % of this age group (8 % of the total population) and have greater risks of requiring hospitalisation, being admitted to ICUs, and of death due to infection; early NAI treatment is assumed to confer a greater benefit for this group than for the general population. Health Care workers comprise 325,000 of the 5–65 age group (informed by expert advice and 2011 census data [15]); they have no significant risk factors [16] but are separated from the general population in order to estimate the impact of pandemic scenarios on the health care work force. General population comprise the remainder of the 5–65 age group and have no significant risk factors.

Each group can be targeted independently for treatment and/or post-exposure prophylaxis; the effects of these interventions on subsequent transmission and on case severity differ by risk group. The model framework used to investigate the effects of targeted NAI strategies combined a mechanistic compartment-model of infection (stratified by risk group) with a finite-capacity compartment model of clinical pathways, subject to likely health care capacities and mean lengths of stay for inpatient settings. We now describe these two models in turn.

### Infection model

The infection model has been described and analysed in previous studies [6, 8]. It is based on a classic susceptible-exposed-infectious-recovered (SEIR) paradigm. All individuals are assumed fully susceptible (S) at the outset of the epidemic, and vulnerable to acquiring infection (E) upon contact with an infectious (I) case. Once recovered (R), individuals are assumed to be fully resistant to reinfection. All simulations commence with 100 infections in the population, distributed between the E and I classes in proportion to the mean duration of the latent and infectious periods.

The model incorporates a dynamic “contact” label, applied to a fixed number of individuals drawn from the whole population each time a new infectious case appears. We define these contacts, based on the findings of sociological studies, as those people who have been sufficiently close to an infected individual to conceivably contract infection. Only contacts of an infectious case may proceed to the exposed and infectious classes, however the majority of contacts escape unscathed, returning to their original state within 72 hrs of exposure. The capture of “contacts” in the model framework allows simulation of targeted post-exposure antiviral prophylaxis (PEP) [17].

In modelling the delivery of antiviral agents to the population, the model accounts for (1) a drop in efficacy due to delays in distribution (e.g., due to the requirement for laboratory confirmation during the Initial Action Stage and (2) constraints on the maximum rate of delivery of antivirals to the population (related to health sector capacity). Both of these factors have previously been shown to dramatically influence the expected outcome from an antiviral intervention and to modify stockpile usage [6]. We assumed that the maximum antiviral delivery ranged from a low estimate of 10^3^ packets per day to an aspirational target of 10^5^ packets per day and drew samples from a log-uniform distribution.

### Clinical pathways model

**Fig. 1:**
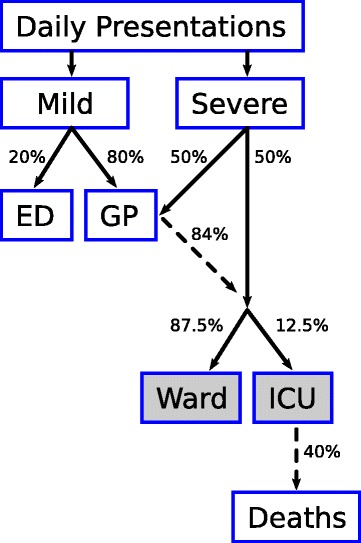
Assumed clinical pathway in the model, reflecting predestined clinical course and potential points of intervention. *Dashed arrows* indicate outflows that are only a fraction of the inflow; percentages shown are for the general population, and the values may differ for other strata (e.g., High-Risk, see Table 3). *Shaded boxes* indicate compartments with residence times greater than one day (i.e., where available capacity is determined by *prevalence*, not *incidence*)

Some proportion of infections will require hospitalisation (“severe cases”) while some proportion of infections will not require hospitalisation and may present to outpatient settings (“mild cases”). The proportion of mild cases that present to hospital EDs rather than to GP clinics in Australia was estimated to be 20 %, based on expert consultation. It is further assumed that a fraction of the cases that will ultimately become severe will present early in their clinical course to an outpatient setting and, should they receive early treatment, their risk of subsequent hospitalisation is reduced [10]. Hospitalised cases have a risk of ICU admission (which varies by risk group and the provision of treatment) and the ICU admissions have a risk of death (which also varies by risk group and the provision of treatment) as shown in Fig. 1.

**Table 1:** Hospital bed and daily consultation capacities for each health care setting

Setting	Total capacity	Available capacity	Mean length of stay
ICU	2,000 [37]	1,000 beds	10 days ^a^
Ward	55,000 [38] ^b^	27,600 beds	5 days [38] ^b^
ED	17,800 [39, 40] ^c^	8,900 consults	—
GP	342,000 [41]	171,000 consults	—

GP consultation capacity was calculated under the assumption that each GP may consult with up to 10 influenza patients per day. Note that we do not account for additional constraints on health sector capacity that may plausibly arise from health care work illness or absenteeism

^a^Assumed the length of stay for ICU cases is double that for other hospitalised cases

^b^Beds in public acute hospitals, mean length of stay for overnight acute separations

^a^Based on annual accident and emergency visits

**Table 2:** Pandemic influenza scenarios, identified by number (“#”)

#	Transmissibility	*R*_0_	Severity	*η*	*α*_*m*_	Mean CAR	Mean AR
1	Low	1.05–1.20	Low	10 ^−4^–10 ^−3^	9.8 %	2.0 %	20.4 %
2	High	1.40–1.70	Low	10 ^−4^–10 ^−3^	9.8 %	6.0 %	61.0 %
3	Low	1.05–1.20	Moderate	10 ^−3^–10 ^−2^	11.6 %	2.3 %	20.4 %
4	Moderate	1.20–1.40	Moderate	10 ^−3^–10 ^−2^	11.6 %	4.8 %	42.0 %
5	High	1.40–1.70	Moderate	10 ^−3^–10 ^−2^	11.6 %	7.0 %	61.0 %
6	Low	1.05–1.20	High	10 ^−2^–10 ^−1^	29.8 %	5.7 %	20.4 %
7	High	1.40–1.70	High	10 ^−2^–10 ^−1^	29.8 %	17.1 %	61.0 %

Note that low transmissibility represents low-level epidemic activity. *η* is the proportion of infections that, in the absence of early treatment, will require hospitalisation (“severe cases”). *α*_*m*_ is the proportion of non-severe infections that present to outpatient settings (“mild cases”). The Clinical Attack Rate (CAR) is the proportion of the population that present due to pandemic influenza infection; the Attack Rate (AR) is the proportion of the population infected during the pandemic.

**Table 3:** Risks of clinical outcomes, with and without NAI treatment, for each stratum (note that “Others” comprises the elderly, health care worker, and general population strata)

		Risk		
Outcome	Precondition(s)	High-risk	Children	Others
Hospitalisation	No Early Rx	1	1	1
Hospitalisation	Early Rx	0.5	1	0.84
ICU admission	Hospitalisation, No Rx	0.395	0.144	0.144
ICU admission	Hospitalisation, Rx	0.25	0.144	0.125
Death	ICU admission, No Rx	0.949	0.461	0.461
Death	ICU admission, Rx	0.6	0.461	0.4

National consultation and admission capacities for each health care setting were informed by public reports of Australian health care infrastructure, under the assumption that in a worst-case scenario up to 50 % of total capacity in each health care setting could possibly be devoted to influenza patients (Table 1).

Patients are admitted to general wards with a mean length of stay of 5 days, and are admitted to ICUs with a mean length of stay of 10 days. Therefore, it is the *prevalence* of cases requiring hospitalisation that determines the available ward and ICU bed capacities for new admissions. Admissions are preferentially allocated by strata, with priority given to health care workers and high-risk adults.

In the event that there is insufficient capacity to admit a newly-presenting case, the following hierarchy of case priorities and overflows are applied:

1. ICU admissions are preferentially allocated in the following order: (1) health care workers; (2) high-risk adults; (3) children; (4) elderly; and (5) general adult population.
2. Any cases that cannot be admitted to an ICU are considered for admission to a general ward, subject to the same order of preferential allocation.
3. In the situation that there is insufficient capacity to admit all cases that require hospitalisation, these cases are assumed to instead present to hospital EDs.
4. A fixed proportion of the mild cases present to hospital EDs, subject to the same order of preference as for ICU and ward admissions.
5. Presentations that cannot receive consultation at an ED are assumed to present at GP clinics.
6. All remaining presentations present at GP clinics, subject to the same order of preference.
7. Presentations that cannot receive a GP consultation are unable to receive antiviral treatment, on the grounds that there was no capacity to consult with these patients.

### Pandemic scenarios

**Fig. 2:**
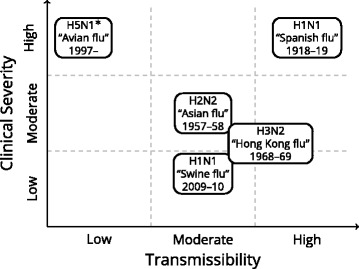
The classification of previous influenza pandemics. Note that the H5N1 avian flu outbreak is not a true pandemic (transmission is sporadic), but is included for illustration

The Australian Health Management Plan for Pandemic Influenza (AHMPPI) defines pandemic impact levels based on the clinical severity of the disease and on the transmissibility of the virus between humans, and characterises both qualities using a “Low”, “Moderate”, “High” scale [11]. Consistent with previous modelling studies and as used in the AHMPPI, we used the pandemic scenarios defined in Table 2. The classification of past influenza pandemics according to these scenario definitions are shown in Fig. 2.

For each scenario, model uncertainties (e.g., epidemic time-course, effectiveness of NAIs) were accounted for by using Latin hypercube sampling (LHS) to randomly select model parameter combinations for 10,000 simulations. We report outcome measures in terms of their median, 5 ^*th*^ and 95 ^*th*^ percentiles, unless stated otherwise.

### Antiviral strategies

For the first four weeks of the epidemic—corresponding to the Initial Action Stage as defined in the AHMPPI—all identified cases are provided with treatment and post-exposure prophylaxis (PEP) is provided to all identifiable contacts. This intensive response has previously been shown necessary to have any chance of significantly reducing infection in those scenarios where such an outcome is possible. This outcome is only achievable in the particular scenario where the disease is both *visible* (i.e., high severity) and has limited transmissibility (i.e., *R*_0_<1.25 [18]) *and* an intensive combined treatment and prophylaxis recommendation is initiated early in the outbreak. Data collected during this stage will allow the outbreak to be classified according to the pandemic scenarios defined above.

The Initial Action Stage is followed by a Targeted Action Stage (for the remaining duration of the epidemic) where treatment and prophylaxis recommendations are revised and targeted for maximum effect as part of a proportionate response for the relevant pandemic scenario. Four alternative targeted strategies for this stage are considered here: Rx All/PEP Eld, HR Treatment of all identified cases regardless of risk stratum or setting of care, and provision of prophylaxis for individuals in the “Elderly” and “High-Risk” strata. Rx All Treatment of all identified cases regardless of risk stratum or setting of care, and no recommendation for prophylaxis. Rx At-Risk, Hosp Treatment of all identified cases in the “Children”, “Elderly”, “High-Risk” and “HCW” strata, and of all cases in hospital and ICU settings. Rx Hosp Treatment of all cases in hospital and ICU settings.

### Effects of antiviral treatment and prophylaxis

The effects of NAI treatment on clinical outcomes for severe cases are listed in Table 3. We assumed that, with the provision of NAIs, high-risk adults were twice as likely to require ICU admission as other adults (0.25 vs 0.125) and were three times more likely to die due to severe infection than other adults [19]. Using the risk ratios for total influenza-related complications reported by Falagas et al. (0.74 for otherwise healthy patients and 0.37 for high-risk patients [20]), we calculated the counter-factual risks of ICU admission and death in the high-risk and general adult populations (refer to the “No Rx” rows of Table 3). On the understanding that NAI treatment confers negligible benefits to children [10, 13, 14], we did not reduce either risk for the children stratum.

A recent population cohort data linkage study of the 2009 H1N1 pandemic in British Columbia reported that the hazard ratio of all-cause hospitalisation, given early NAI treatment, was approximately 0.84 in the general population and 0.52 among those with co-morbidity [10]. We assumed that the relative risk of hospitalisation given early NAI treatment was identical to these hazard ratios: 0.52 for the high-risk stratum, 0.84 for all other adult strata, and 1.0 for children (assuming that NAI treatment confers no benefits to children). In the absence of early treatment, all severe cases require hospitalisation.

These assumptions are also consistent with a recent world-wide meta-analysis of patients hospitalised with A(H1N1)pdm09, which reported that early NAI treatment, in comparison to late NAI treatment, was associated with significant reductions in mortality and likelihood of requiring ventilatory support [14].

## Results

**Fig. 3:**
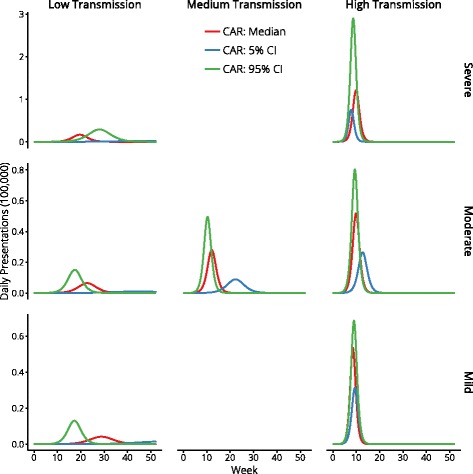
Representative epidemic curves for each pandemic scenario, selected by Clinical Attack Rate (CAR)

For each of the seven pandemic scenarios we performed 10,000 simulations with different combinations of parameter values, meaning that no single epidemic can characterise any of these scenarios. This design allows us to account for uncertainty in the precise nature of the epidemic itself, in the early (imprecise) estimates of transmissibility and severity obtained during initial action, in the effectiveness of NAIs to reduce susceptibility, pathogenicity, and transmission, in logistic capacities that limit NAI distribution, and in population compliance. An illustration of the variety of epidemics that were considered in this study are shown in Fig. 3, which depicts the median, 5 ^*th*^ and 95 ^*th*^ percentile epidemic curves, as measured by Clinical Attack Rate (CAR). The epidemic duration for each scenario is reported in Table 4.

**Table 4:** Epidemic duration for each pandemic scenario, reported as the interval over which 90 % of all infections occurred

	Epidemic duration (weeks)		
Scenario	Median	(5^*th*^, 95^*th*^ % iles)	Mean
Low transmission	18.4	(10.9, 26.5)	18.8
Medium transmission	8.1	(5.5, 12.3)	8.4
High transmission	4.7	(3.3, 6.8)	4.8

**Fig. 4:**
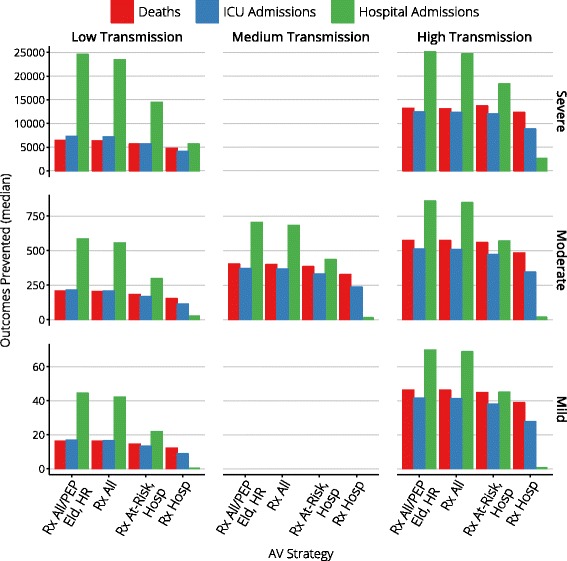
Median clinical outcomes for each antiviral strategy

**Fig. 5:**
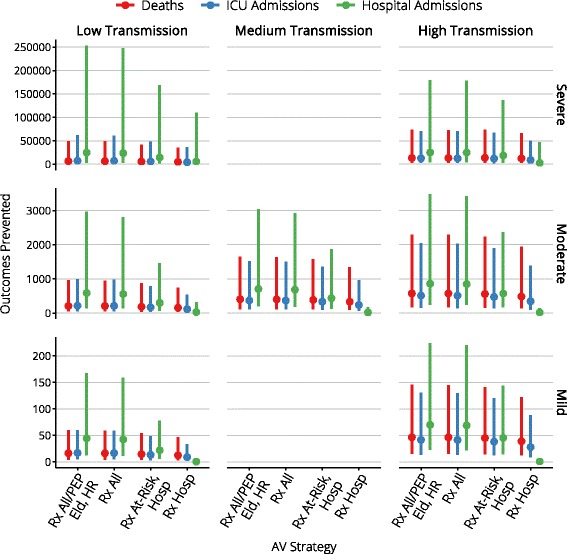
The range of clinical outcomes for each antiviral strategy

**Fig. 6:**
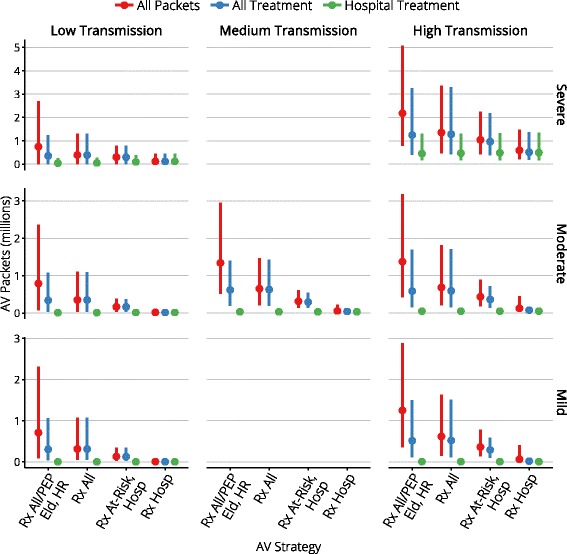
The range of stockpile usage for each antiviral strategy

**Fig. 7:**
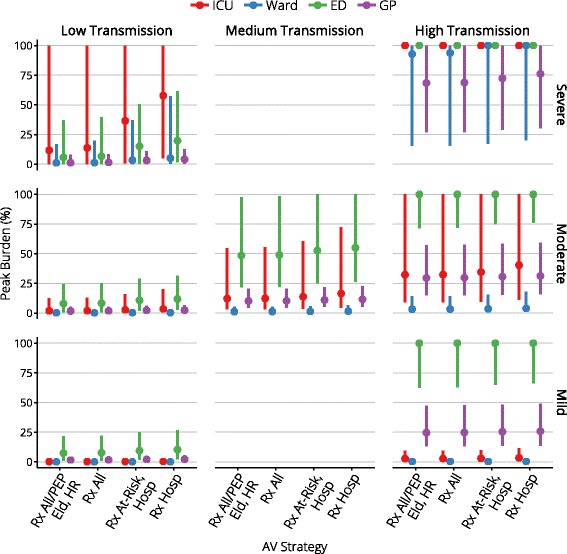
The peak burden on each health care setting

**Fig. 8:**
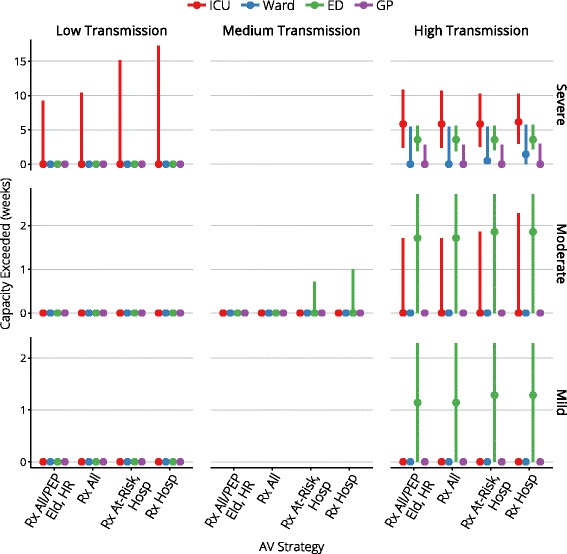
The duration for which capacity is exceeded, for each health care setting

Figures 4, 5, 6, 7, and 8 present an overview of the key health care outcomes for each of the pandemic scenarios and for each targeted antiviral strategy for the Targeted Action Stage. Here, we identify the key findings for each of the following pandemic scenarios: #4 (moderate severity, medium transmissibility), #6 (high severity, low transmissibility) and #7 (high severity, high transmissibility).

### Moderate severity, medium transmissibility

Medium transmission scenarios are not controllable by antiviral interventions, regardless of the proportion of infections that are identifiable. In these scenarios antiviral interventions have only a marginal effect on the prevalence of infection, but can yield reductions in case severity, hospital admissions, and deaths.

Across the range of antiviral strategies for the Targeted Action Stage, the median peak ICU occupancy ranges from 119–164 (out of 1000 beds) and the median peak ward occupancy ranges from 317–420 (out of 27,600 beds), indicating that there is more than sufficient hospital capacity to care for all cases that might require hospitalisation. This holds true even for the 95th percentiles (540–723 ICU beds and 1412–1824 ward beds).

While some of the more conservative antiviral strategies cause ED capacity to be overwhelmed (in a small fraction of the simulations) by 3–7 % for a period of up to one week, this is a direct result of assuming that 1 in 5 mild presentations occurs at an ED rather than at a GP clinic. We note here that this assumption was based on expert consultation and it is unclear how to validate it against available public health data. The peak burden on GPs, on the other hand, only consumes up to 22 % of the available consultation capacity. The conclusion to draw from these observations is that there is more than sufficient capacity for all mild cases to receive consultation, but that the ED consultation capacity can be temporarily overwhelmed if a moderate proportion of these cases elect to present to EDs rather than to GP clinics.

The more liberal antiviral strategies produce median reductions of 400 fewer deaths, 370 fewer ICU admissions, and 700 fewer hospitalisations, given median stockpile usage of 650,000 to 1.3 million doses (95th percentile usage is less than 3 million doses). More conservative strategies produce median reductions of 330–380 fewer deaths, 235–330 fewer ICU admissions, and 15–427 few hospitalisations, given median stockpile usage of 60,000 to 300,000 doses (95th percentile usage is less than 600,000 doses).

### High severity, low transmissibility

Low transmission scenarios are more readily controllable if a sufficient proportion of infections are identifiable and can be provided with antivirals for treatment or prophylaxis [18]. This visibility substantially reduces the clinical attack rate (CAR) and can, in ideal circumstances, lead to successful mitigation of the epidemic.

For this scenario, the absence of antiviral interventions results in median CARs of 6.8 % in the High-Risk population and 4.9 % for the rest of the population. In contrast, the application of a 4 week Initial Action Stage followed by a Targeted Action Stage where antivirals are only provided to hospitalised cases results in median CARs of 5.5 % in the High-Risk population and 4.1 % for the rest of the population. As the antiviral strategy for the Targeted Action Stage becomes more liberal, the median CARs are reduced even further, to minimums of 0.9 % in the High-Risk population and 0.7 % for the rest of the population. The most liberal strategy (“Rx All/PEP Eld, HR”) substantially improves the relative risk of presentation (median relative risk: 0.376), hospital admission (median relative risk: 0.320), ICU admission (median relative risk: 0.223) and death (median relative risk: 0.146); see the summary tables in Additional file 1 for further details. This control is achieved with a median stockpile usage of fewer than one million doses for the most liberal antiviral strategies, where the majority of the doses are used for post-exposure prophylaxis.

Overall hospital capacity is never exceeded. ICU capacity is exceeded in a small number of simulations, but the mean and median number of days for which this occurs is zero. With even the most conservative antiviral strategy during the Targeted Action Stage, the mean and median peak ICU occupancy is 577 beds (out of 1000 beds), and the mean and median peak ward occupancy is 1394 beds (out of 27,600 beds).

The clearest indicators of the effect that antiviral interventions have on the epidemic burden are the reductions in deaths, ICU admissions and hospitalisations that are achieved, in comparison to the same scenario with no antiviral interventions. When the most conservative strategy is used during the Targeted Action Stage, and antivirals are only provided for treatment to hospitalised cases, the median reductions are 4,780 fewer deaths, 4,095 fewer ICU admissions and 5,656 fewer hospital admissions. These outcomes improve as more liberal antiviral strategies are used during the Targeted Action Stage, to median reductions of 6,437 fewer deaths, 7,793 fewer ICU admissions and 24,566 fewer hospital admissions.

### High severity, high transmissibility

High transmission scenarios are not controllable by antiviral interventions, regardless of the proportion of infections that are identifiable. In these scenarios antiviral interventions cannot affect the prevalence of infection, but can yield reductions in case severity, hospital admissions, and deaths. Compared with the medium transmission scenarios, antiviral interventions will achieve greater absolute reductions in burden, but the higher transmission means that these are smaller fractional reductions in burden.

In this worst-case scenario, ICU bed capacity is exceeded for 6 weeks and ED consultation capacity is exceeded for 3–4 weeks, regardless of the antiviral strategy for the Targeted Action Stage (i.e., essentially for the duration of the pandemic). The more liberal antiviral strategies produce median reductions of 13,000 fewer deaths, 12,400 fewer ICU admissions, and 25,000 fewer hospitalisations, given median stockpile usage of 1.3 to 2.2 million doses (95th percentile usage is 5 million doses). More conservative strategies produce median reductions of 12,300–13,700 fewer deaths, 8,800–12,000 fewer ICU admissions, and 2,600–18,200 few hospitalisations, given median stockpile usage of 600,000 to 1 million doses (95th percentile usage is 2.2 million doses).

All choices of antiviral strategy for the Targeted Action Stage reduce deaths by approximately 50 % and also reduce ICU and hospital admissions, but while the proportional reduction in clinical burden is similar to the medium transmission and moderate severity scenario (above), the absolute burden remains substantially higher. In this case, the most important conclusions to draw are that antiviral strategies can substantially reduce the number of deaths in even the most severe pandemic scenarios where hospital bed capacities are substantially overwhelmed for many weeks, *assuming that antivirals can continue to be delivered in a timely and effective manner* (as they are through general practice in the model).

### Stockpile consumption

Stockpile consumption for the intensive response delivered in the Initial Action Stage is shown in Table 5 for each pandemic scenario. The salient detail is that delivering this response consumes a small number of doses, relative to the size of a national stockpile, even for the 95 ^*th*^ percentile *across all scenarios*.

**Table 5:** Initial Action Stage stockpile consumption — median (95 ^*th*^ percentile)

	Low transmission	Moderate transmission	High transmission
High severity	2,400 (5,300)		43,000 (330,000)
Moderate severity	2,500 (6,000)	9,000 (170,000)	53,000 (380,000)
Low severity	2,500 (6,800)		56,000 (400,000)

Overall stockpile consumption for each scenario and each targeted antiviral strategy is shown in Fig. 6. Recall that the (imposed) maximum distribution rate varied from 10^3^ to 10^5^ packets per day. In the worst-case scenario (high severity, high transmissibility) and with large maximum distribution rates (e.g., at least 9×10^4^ packets per day), the maximum rate was reached in two-thirds of the simulations with liberal treatment of cases in the community (the “Rx All/PEP Eld, HR” and “Rx All” strategies).

It is clearly evident that the most liberal strategy (“Rx All/PEP Eld, HR”) consumes a substantially greater number of treatment courses than all other strategies, due to the provision of post-exposure prophylaxis to all elderly and high-risk contacts, but generally confers no significant benefit over the more conservative “Rx All” strategy. The targeted treatment strategies (“Rx At-Risk, Hosp” and “Rx Hosp”) consume even fewer treatment courses than the “Rx All” strategy but, as identified above, at the cost of preventing substantially fewer hospital admissions.

## Discussion

### Principal findings

NAI treatment strategies that allow liberal distribution of antivirals for early treatment in outpatient settings yielded the greatest benefits, evidenced by reductions in hospitalisations, critical care requirements and deaths, for *all* of the considered pandemic scenarios. Restriction of community-based treatment to risk groups is effective in those groups, but fails to prevent the large proportion of cases arising from lower risk individuals who comprise the majority of the population. In even the most severe scenarios, median stockpile consumption for treatment was 1.3 million doses, sufficient to cover 6.5 % of the population (95 ^*th*^ percentile: 3.3 million doses, sufficient to cover 16.5 % of the population).

In high severity scenarios, we predict that capacity constraints within ICUs and hospitals will be exceeded, placing greater pressure on community-based health services. NAI treatment strategies can substantially reduce the number of deaths in even the most severe pandemic scenarios where hospital bed capacities are substantially overwhelmed for many weeks, assuming that antivirals can continue to be delivered in a timely and effective manner (as they are through general practice in the model). While we have assumed that general practitioners will be able to deliver effective health care to the resulting “overflow” population, more consideration of models of care delivery in such situations is needed to ensure access to needed services.

### Study strengths and weaknesses

Strengths include our use of LHS sampling and broad parameter ranges to consider a wide range of epidemics, logistic capacities, and compliance, for each of the pandemic scenarios; the spread of the results for each scenario (reported as 5th and 95th percentiles) is therefore indicative of the impact and outcomes, without being strongly tied to specific values of model parameters.

The major weaknesses are: (a) the assumption that up to 50 % of actual health care capacities can be devoted to treating influenza patients (approaching or exceeding these capacities therefore represents an “apocalpytic” pandemic, with broader ramifications for society as a whole); (b) that the health care workforce does not suffer from fatigue, absenteeism, or depletion due to illness (any of which would compromise intervention delivery, and consultation and bed capacities); (c) the role of emergency departments as “gateways” to the hospital system is not explicitly configured, including the potential for overwhelmed EDs to constrain capacity for hospital admissions; and (d) multiple presentations for a single case is only considered for severe cases who receive (ineffective) early treatment and subsequently require hospital admission, thus excluding the possibility of repeat presentations in general *despite* evidence that this may be a common occurrence [21].

Critically, the effectiveness of NAIs (see Table 3) is based on available evidence, which is mostly observational since there are no clinical trial data in hospitalised patients and in high-risk groups [9]. The recent review by the Academy of Medical Sciences and the Wellcome Trust states that “there is a lack of evidence to guide decisions on NAI treatment for high-risk groups and children,” but also notes that “the steering group does not support the assumption that observational data are invariably of less use than data from RCTs” [9]. The interpretation of the available evidence, however, remains a subject of some controversy.

We have not accounted for social distancing measures such as school closures in this study. Despite the lack of substantial quantitative evidence to inform mathematical models and optimal implementation [22], and concerns about societal costs [23, 24], such methods have been widely used in past epidemics (e.g., in the 2009 pandemic in Australia [25] and other countries [26], and in Australia during the 1918–19 pandemic [27]). Evidence from 2009 in the state of Victoria, Australia, is that compliance with behavioural and pharmaceutical recommendations was high, but were unlikely to have substantially altered the course of the epidemic [25]. Due to the timing and circumstances, this likely reflects a “best case” estimate of public compliance during a moderate to severe influenza pandemic [25].

These limitations highlight the critical importance of considering tailored models of care delivery and how appropriate communication may influence health care seeking behaviour in target populations.

The timing of the pandemic waves with respect to season is another factor that can impact the basic reproduction number, causing substantial differences in the pandemic impact experienced in different regions of the world and confounding the applicability of overseas pathogen assessments for the local context. This reinforces the importance of having local or region-specific real-time surveillance and *R*_0_ estimation protocols in place.

We have also restricted this study to considering responses to a single pandemic wave. Multiple waves were observed in 2009 in the UK [28, 29] and Australia [30, 31], in 1918–19 in the UK [32–34] and Australia [27], and are typical of all pandemics in the 20th century [35]. Accounting for secondary and tertiary waves is an integral part of pandemic preparedness, which we have not addressed here; implementation of a strain-specific vaccine is identified as the definitive measure to reduce morbidity and mortality in subsequent pandemic waves in an Australian pandemic response [11]. In subsequent waves there may be greater knowledge about the pathogen, but external factors such as seasonality, vaccine availability [31], social behaviour, and changing host immunity [33, 34] may act as confounders and greatly affect the impact of these subsequent waves. All of these issues are clearly of real importance, but are beyond the scope of this study.

### Meaning and implications

The results of this study suggest that the optimal use of an antiviral stockpile in the event of an influenza pandemic is to provide treatment to as many cases as possible, in both inpatient and outpatient settings. In the unlikely scenario that the pandemic strain exhibits both high clinical severity and low transmissibility, exhaustive contact-tracing and provision of post-exposure prophylaxis may be able to sufficiently reduce transmission as to mitigate the pandemic. In all other scenarios, this outcome is not achievable and the provision of post-exposure prophylaxis both confers no benefits to the population and substantially increases stockpile consumption. This finding highlights the critical importance of intensive early data gathering to inform impact assessment (e.g., based on results of “first few hundred” studies [36]), enabling reorientation of public health efforts for proportionate and effective response.

## Conclusions

The key issue identified in this study is the importance of understanding how different models of care may enable the delivery effective interventions in the event of a pandemic, without overwhelming existing day-to-day and surge capacities. We have shown that a modest NAI stockpile permits liberal (early) treatment in both outpatient and inpatient settings, which can substantially reduce hospitalisations, critical care requirements and deaths in the event of an influenza pandemic. However, these outcomes are only achievable if liberal treatment strategies can be effectively deployed within the constraints of the existing health care infrastructure.

## Additional file

Additional file 1 Model description and additional results tables. This document presents the model equations, the distributions from which the model parameters were sampled, and tables of simulation results for each pandemic scenario and for each targeted NAI strategy. (PDF 103 kb)

## Abbreviations

AHMPPI: Australian Health Management Plan for Pandemic Influenza
CAR: Clinical attack rate
ED: Emergency department
GP: General practice
HCW: Health care worker
HR: High Risk population stratum, aged 5-65 years and possessing underlying risk conditions
ICU: Intensive care unit
LHS: Latin hypercube sampling
NAI: Neuraminidase inhibitor
PEP: Post-exposure prophylaxis
RCT: Randomised controlled trial
Rx All/PEP Eld, HR: Targeted NAI strategy: treatment of all identified cases regardless of risk stratum or setting of care, and provision of prophylaxis for individuals in the “Elderly” and “High-Risk” strata
Rx All: Targeted NAI strategy: treatment of all identified cases regardless of risk stratum or setting of care, and no recommendation for prophylaxis
Rx At-Risk, Hosp: Targeted NAI strategy: treatment of all identified cases in the “Children”, “Elderly”, “High-Risk” and “HCW” strata, and of all cases in hospital and ICU settings
Rx Hosp: Targeted NAI strategy: treatment of all cases in hospital and ICU settings
SEIR: Susceptible-exposed-infectious-recovered compartment model
