# Supplementary material for: Reducing disease burden in an influenza pandemic by targeted delivery of neuraminidase inhibitors: mathematical models in the Australian context
Source: BMC Infect Dis. 2016 Oct 10;16:552. doi: 10.1186/s12879-016-1866-7 (PMC5057455; doi:10.1186/s12879-016-1866-7)
Supplement: Additional file 1 — Model description and additional results tables. This document presents the model equations, the distributions from which the model parameters were sampled, and tables of simulation results for each pandemic scenario and for each targeted NAI strategy. (PDF 103 kb) [file 12879_2016_1866_MOESM1_ESM.pdf]

S1 Tables of results

To aid interpretation, we have colour-coded each row in the following tables by *pandemic scenario*, according to the colour scheme shown below. Green represents low transmission (Scenarios 1, 3, 6), blue represents medium transmission (Scenario 4) and red represents high transmission (Scenarios 2, 5, 7), while the colour intensity represents the pandemic severity.

**Scenario 6**<br>**Low transmission**<br>**High severity**		**Scenario 7**<br>**High transmission**<br>**High severity**
**Scenario 3**<br>**Low transmission**<br>**Moderate severity**	**Scenario 4**<br>**Medium transmission**<br>**Moderate severity**	**Scenario 5**<br>**High transmission**<br>**Moderate severity**
**Scenario 1**<br>**Low transmission**<br>**Mild severity**		**Scenario 2**<br>**High transmission**<br>**Mild severity**

Table S1: The colour scheme used to identify each pandemic scenario.

We report outcome measures in terms of their median, 5th and 95th percentiles, and arithmetic mean, for each of the Targeted Action Stage strategies:

1: Rx All/PEP Eld, HR Treatment of all identified cases regardless of risk stratum or setting of care, and provision of prophylaxis for individuals in the "Elderly" and "High-Risk" strata.

2: Rx All Treatment of all identified cases regardless of risk stratum or setting of care, and no recommendation for prophylaxis.

3: Rx At-Risk, Hosp Treatment of all identified cases in the "Children", "Elderly", "High-Risk" and "HCW" strata, and of all cases in hospital and ICU settings.

4: Rx Hosp Treatment of all cases in hospital and ICU settings.

Scenario	Strategy	High-Risk	Others
Low Tx/ Mild	1	1.7% (0.2%, 3.1%) 1.6%	1.7% (0.2%, 3.1%) 1.6%
Low Tx/ Mild	2	1.7% (0.2%, 3.1%) 1.7%	1.7% (0.2%, 3.1%) 1.7%
Low Tx/ Mild	3	1.9% (0.5%, 3.3%) 1.9%	1.9% (0.5%, 3.3%) 1.9%
Low Tx/ Mild	4	2.0% (0.6%, 3.5%) 2.0%	2.0% (0.6%, 3.4%) 2.0%
Low Tx/ Mod	1	2.0% (0.2%, 3.9%) 2.0%	1.8% (0.1%, 3.5%) 1.8%
Low Tx/ Mod	2	2.1% (0.2%, 4.0%) 2.1%	1.9% (0.2%, 3.6%) 1.9%
Low Tx/ Mod	3	2.4% (0.5%, 4.4%) 2.4%	2.2% (0.5%, 3.8%) 2.2%
Low Tx/ Mod	4	2.5% (0.7%, 4.5%) 2.6%	2.3% (0.6%, 4.0%) 2.3%
Low Tx/ Sev	1	2.1% (0.0%, 9.3%) 3.0%	1.6% (0.0%, 6.6%) 2.2%
Low Tx/ Sev	2	2.5% (0.0%, 9.9%) 3.3%	1.8% (0.0%, 6.9%) 2.4%
Low Tx/ Sev	3	4.7% (0.1%, 12.5%) 5.2%	3.5% (0.1%, 9.0%) 3.7%
Low Tx/ Sev	4	5.5% (0.4%, 14.3%) 6.2%	4.1% (0.3%, 10.3%) 4.5%
Med Tx/ Mod	1	4.9% (3.1%, 7.6%) 5.1%	4.4% (2.8%, 6.7%) 4.6%
Med Tx/ Mod	2	5.0% (3.1%, 7.7%) 5.1%	4.5% (2.8%, 6.7%) 4.6%
Med Tx/ Mod	3	5.1% (3.2%, 7.9%) 5.3%	4.6% (2.9%, 6.9%) 4.7%
Med Tx/ Mod	4	5.2% (3.3%, 8.0%) 5.4%	4.7% (3.0%, 7.0%) 4.8%
High Tx/ Mild	1	5.9% (3.9%, 8.1%) 5.9%	5.8% (3.9%, 8.1%) 5.9%
High Tx/ Mild	2	5.9% (3.9%, 8.2%) 6.0%	5.8% (3.9%, 8.1%) 5.9%
High Tx/ Mild	3	6.0% (4.0%, 8.2%) 6.0%	5.9% (3.9%, 8.1%) 5.9%
High Tx/ Mild	4	6.0% (4.0%, 8.3%) 6.1%	5.9% (3.9%, 8.2%) 6.0%
High Tx/ Mod	1	7.5% (4.9%, 11.1%) 7.7%	6.8% (4.4%, 9.7%) 6.9%
High Tx/ Mod	2	7.5% (4.9%, 11.1%) 7.7%	6.8% (4.4%, 9.7%) 6.9%
High Tx/ Mod	3	7.6% (4.9%, 11.2%) 7.8%	6.8% (4.5%, 9.8%) 7.0%
High Tx/ Mod	4	7.7% (5.0%, 11.3%) 7.8%	6.9% (4.5%, 9.9%) 7.0%
High Tx/ Sev	1	19.9% (10.2%, 40.5%) 22.1%	14.3% (7.6%, 30.0%) 16.1%
High Tx/ Sev	2	20.0% (10.3%, 40.6%) 22.2%	14.3% (7.7%, 30.0%) 16.1%
High Tx/ Sev	3	20.4% (10.4%, 41.0%) 22.6%	14.6% (7.7%, 30.4%) 16.3%
High Tx/ Sev	4	20.6% (10.5%, 41.6%) 22.9%	14.8% (7.8%, 30.7%) 16.6%

Table S2: The Clinical Attack Rate (CAR), reported for the High-Risk stratum and for all other strata combined ("Others"). **Key message:** Except in the low-transmission high-severity scenario (dark green), the choice of strategy for the Targeted Action Phase has minimal effect on the Clinical Attack Rate.

Scenario	Strategy	Net Usage (100,000s)	Hospital Rx (100,000s)
Low Tx/ Mild	1	7.11 (0.93, 23.18) 9.24	1.2 (0.1, 5.0) 1.7
Low Tx/ Mild	2	3.14 (0.48, 10.74) 4.26	1.2 (0.1, 5.0) 1.8
Low Tx/ Mild	3	1.24 (0.27, 3.27) 1.51	1.4 (0.3, 5.4) 2.0
Low Tx/ Mild	4	0.04 (0.02, 0.11) 0.06	1.5 (0.4, 5.6) 2.1
Low Tx/ Mod	1	7.90 (0.74, 23.69) 9.67	11.1 (1.0, 46.8) 15.9
Low Tx/ Mod	2	3.51 (0.38, 10.99) 4.49	11.5 (1.1, 47.9) 16.4
Low Tx/ Mod	3	1.60 (0.31, 3.60) 1.76	13.9 (2.9, 52.5) 19.2
Low Tx/ Mod	4	0.18 (0.05, 0.61) 0.24	15.1 (3.6, 54.7) 20.5
Low Tx/ Sev	1	7.48 (0.03, 26.99) 9.29	43.9 (0.2, 262.9) 72.5
Low Tx/ Sev	2	3.92 (0.03, 13.02) 4.64	51.4 (0.2, 286.6) 80.4
Low Tx/ Sev	3	2.91 (0.08, 7.75) 3.24	96.7 (2.7, 391.0) 132.3
Low Tx/ Sev	4	1.20 (0.11, 4.53) 1.63	117.0 (9.8, 448.4) 160.0
Med Tx/ Mod	1	13.42 (5.16, 29.48) 14.86	32.2 (11.2, 94.9) 40.3
Med Tx/ Mod	2	6.52 (2.10, 14.66) 7.22	32.8 (11.5, 95.6) 40.9
Med Tx/ Mod	3	3.03 (1.43, 5.88) 3.26	33.8 (11.9, 98.1) 42.3
Med Tx/ Mod	4	0.57 (0.18, 2.19) 0.78	34.5 (12.1, 99.8) 43.0
High Tx/ Mild	1	12.51 (3.57, 28.85) 13.88	4.9 (1.8, 14.1) 6.1
High Tx/ Mild	2	6.18 (1.54, 16.36) 7.25	5.0 (1.8, 14.1) 6.2
High Tx/ Mild	3	3.50 (1.30, 7.58) 3.82	5.0 (1.8, 14.3) 6.3
High Tx/ Mild	4	0.63 (0.16, 4.06) 1.16	5.0 (1.8, 14.4) 6.3
High Tx/ Mod	1	13.75 (4.21, 31.76) 15.26	48.9 (17.5, 139.5) 60.9
High Tx/ Mod	2	6.86 (2.04, 18.15) 8.16	49.5 (17.7, 140.3) 61.6
High Tx/ Mod	3	4.24 (1.74, 8.59) 4.57	50.1 (17.9, 142.1) 62.3
High Tx/ Mod	4	1.27 (0.41, 4.46) 1.67	50.4 (18.0, 143.3) 62.8
High Tx/ Sev	1	21.81 (7.85, 50.82) 24.83	454.4 (167.6, 1298.2) 562.8
High Tx/ Sev	2	13.55 (4.75, 33.58) 15.51	471.1 (174.4, 1309.7) 578.3
High Tx/ Sev	3	10.28 (4.22, 22.10) 11.36	487.2 (177.5, 1327.1) 594.1
High Tx/ Sev	4	5.94 (2.19, 14.66) 6.94	494.8 (178.9, 1355.6) 607.0

Table S3: Stockpile usage is reported in terms of the total number of packets that were distributed for treatment of community and hospital presentations and for post-exposure prophylaxis ("Net Usage") and also by the number of packets that were used to treat hospitalised cases ("Hospital Rx"). **Key message:** Liberal antiviral use in the community ("Rx All/PEP Eld, HR" and "Rx All" strategies) increases the net stockpile consumption but can decrease hospital consumption in the high-severity scenarios. Prophylaxis makes a substantial additional drain on the stockpile.

Scenario	Strategy	Outpatient Flu (100,000s)	Outpatient Non-Flu (100,000s)
Low Tx/ Mild	1	2.00 (0.26, 4.28) 2.08	0.00 (0.00, 7.77) 2.05
Low Tx/ Mild	2	2.04 (0.29, 4.36) 2.13	0.00 (0.00, 7.79) 2.08
Low Tx/ Mild	3	0.79 (0.19, 1.53) 0.82	0.00 (0.00, 2.32) 0.72
Low Tx/ Mild	4	0.00 (0.00, 0.01) 0.00	0.00 (0.00, 0.00) 0.00
Low Tx/ Mod	1	2.11 (0.20, 4.69) 2.21	0.00 (0.00, 7.57) 1.99
Low Tx/ Mod	2	2.16 (0.23, 4.79) 2.27	0.00 (0.00, 7.60) 2.02
Low Tx/ Mod	3	0.87 (0.19, 1.71) 0.90	0.00 (0.00, 2.27) 0.72
Low Tx/ Mod	4	0.00 (0.00, 0.01) 0.00	0.00 (0.00, 0.00) 0.00
Low Tx/ Sev	1	1.55 (0.01, 6.39) 2.12	0.00 (0.00, 6.53) 1.44
Low Tx/ Sev	2	1.75 (0.01, 6.72) 2.29	0.00 (0.00, 6.64) 1.52
Low Tx/ Sev	3	1.20 (0.02, 2.94) 1.27	0.00 (0.00, 2.21) 0.71
Low Tx/ Sev	4	0.00 (0.00, 0.01) 0.00	0.00 (0.00, 0.00) 0.00
Med Tx/ Mod	1	3.83 (0.83, 8.16) 4.01	2.22 (0.00, 6.12) 2.43
Med Tx/ Mod	2	3.88 (0.84, 8.28) 4.05	2.27 (0.00, 6.18) 2.48
Med Tx/ Mod	3	1.60 (0.69, 2.92) 1.67	1.06 (0.00, 2.13) 1.00
Med Tx/ Mod	4	0.01 (0.00, 0.03) 0.01	0.00 (0.00, 0.33) 0.06
High Tx/ Mild	1	3.07 (0.53, 9.39) 3.84	2.00 (0.00, 5.78) 2.41
High Tx/ Mild	2	3.11 (0.54, 9.44) 3.88	2.04 (0.00, 5.82) 2.44
High Tx/ Mild	3	1.78 (0.50, 3.57) 1.84	1.11 (0.00, 2.35) 1.16
High Tx/ Mild	4	0.05 (0.02, 0.26) 0.09	0.00 (0.00, 0.58) 0.14
High Tx/ Mod	1	3.17 (0.55, 10.24) 4.10	2.05 (0.18, 6.19) 2.54
High Tx/ Mod	2	3.20 (0.55, 10.34) 4.14	2.10 (0.33, 6.24) 2.58
High Tx/ Mod	3	1.91 (0.51, 3.98) 2.00	1.18 (0.27, 2.54) 1.24
High Tx/ Mod	4	0.05 (0.01, 0.24) 0.08	0.00 (0.00, 0.55) 0.13
High Tx/ Sev	1	3.71 (0.63, 15.89) 5.52	2.32 (0.49, 9.04) 3.28
High Tx/ Sev	2	3.82 (0.64, 16.14) 5.65	2.39 (0.53, 9.17) 3.37
High Tx/ Sev	3	2.59 (0.58, 7.44) 3.08	1.54 (0.39, 4.22) 1.81
High Tx/ Sev	4	0.03 (0.01, 0.19) 0.06	0.00 (0.00, 0.48) 0.11

Table S4: Outpatient stockpile usage is reported in terms of the number of treatment packets that were distributed to persons infected with pandemic influenza ("Outpatient Flu") and to persons not infected with pandemic influenza but presenting with ILI ("Outpatient Non-Flu"), who may receive treatment when a syndromic indication is considered sufficient for initiation of treatment. **Key message:** When using syndromic indication to initiate treatment in the community, the number of doses provided to patients that are not infected with pandemic influenza is less than the number used for effective treatment in the community.

Scenario	Strategy	Treatment (100,000s)	Net (100,000s)
Low Tx/ Mild	1	0.00 (0.00, 0.01) 0.01	0.03 (0.01, 0.07) 0.04
Low Tx/ Mild	2	0.00 (0.00, 0.01) 0.01	0.03 (0.01, 0.07) 0.04
Low Tx/ Mild	3	0.00 (0.00, 0.01) 0.01	0.03 (0.01, 0.07) 0.04
Low Tx/ Mild	4	0.00 (0.00, 0.01) 0.01	0.03 (0.01, 0.07) 0.04
Low Tx/ Mod	1	0.00 (0.00, 0.01) 0.01	0.02 (0.01, 0.06) 0.04
Low Tx/ Mod	2	0.00 (0.00, 0.01) 0.01	0.02 (0.01, 0.06) 0.04
Low Tx/ Mod	3	0.00 (0.00, 0.01) 0.01	0.02 (0.01, 0.06) 0.04
Low Tx/ Mod	4	0.00 (0.00, 0.01) 0.01	0.02 (0.01, 0.06) 0.04
Low Tx/ Sev	1	0.00 (0.00, 0.01) 0.00	0.02 (0.01, 0.05) 0.03
Low Tx/ Sev	2	0.00 (0.00, 0.01) 0.00	0.02 (0.01, 0.05) 0.03
Low Tx/ Sev	3	0.00 (0.00, 0.01) 0.00	0.02 (0.01, 0.05) 0.03
Low Tx/ Sev	4	0.00 (0.00, 0.01) 0.00	0.02 (0.01, 0.05) 0.03
Med Tx/ Mod	1	0.01 (0.01, 0.35) 0.07	0.09 (0.03, 1.73) 0.35
Med Tx/ Mod	2	0.01 (0.01, 0.35) 0.07	0.09 (0.03, 1.73) 0.35
Med Tx/ Mod	3	0.01 (0.01, 0.35) 0.07	0.09 (0.03, 1.73) 0.35
Med Tx/ Mod	4	0.01 (0.01, 0.35) 0.07	0.09 (0.03, 1.73) 0.35
High Tx/ Mild	1	0.11 (0.02, 0.70) 0.23	0.56 (0.11, 4.01) 1.10
High Tx/ Mild	2	0.11 (0.02, 0.70) 0.23	0.56 (0.11, 4.01) 1.10
High Tx/ Mild	3	0.11 (0.02, 0.70) 0.23	0.56 (0.11, 4.01) 1.10
High Tx/ Mild	4	0.11 (0.02, 0.70) 0.23	0.56 (0.11, 4.01) 1.10
High Tx/ Mod	1	0.10 (0.02, 0.68) 0.22	0.53 (0.11, 3.83) 1.05
High Tx/ Mod	2	0.10 (0.02, 0.68) 0.22	0.53 (0.11, 3.83) 1.05
High Tx/ Mod	3	0.10 (0.02, 0.68) 0.22	0.53 (0.11, 3.83) 1.05
High Tx/ Mod	4	0.10 (0.02, 0.68) 0.22	0.53 (0.11, 3.83) 1.05
High Tx/ Sev	1	0.08 (0.01, 0.60) 0.18	0.43 (0.09, 3.32) 0.89
High Tx/ Sev	2	0.08 (0.01, 0.60) 0.18	0.43 (0.09, 3.32) 0.89
High Tx/ Sev	3	0.08 (0.01, 0.60) 0.18	0.43 (0.09, 3.32) 0.89
High Tx/ Sev	4	0.08 (0.01, 0.60) 0.18	0.43 (0.09, 3.32) 0.89

Table S5: Initial Action Phase stockpile usage is reported in terms of the number of packets that were distributed for treatment ("Treatment") and in total ("Net"). **Key message:** Stockpile consumption in the Initial Action Phase is very low in all pandemic scenarios, compared to the net consumption over the course of the entire epidemic (previous tables), even when the number of prophylaxis courses greatly exceeds the number of treatment courses.

Scenario	Strategy	Peak ICU Utilisation	Peak Ward Utilisation
Low Tx/ Mild	1	2 (0, 10) 3	5 (0, 25) 7
Low Tx/ Mild	2	2 (0, 10) 3	5 (0, 26) 8
Low Tx/ Mild	3	2 (0, 12) 4	6 (1, 32) 10
Low Tx/ Mild	4	3 (0, 14) 5	7 (1, 37) 12
Low Tx/ Mod	1	18 (1, 123) 34	48 (4, 318) 87
Low Tx/ Mod	2	19 (2, 128) 35	50 (4, 329) 91
Low Tx/ Mod	3	26 (4, 159) 46	70 (10, 414) 120
Low Tx/ Mod	4	34 (5, 199) 58	85 (13, 493) 145
Low Tx/ Sev	1	117 (1, 1000) 263	295 (3, 4661) 1033
Low Tx/ Sev	2	138 (1, 1000) 286	346 (4, 5396) 1171
Low Tx/ Sev	3	372 (10, 1000) 475	950 (27, 10367) 2390
Low Tx/ Sev	4	578 (49, 1000) 588	1394 (120, 15828) 3642
Med Tx/ Mod	1	122 (31, 545) 182	322 (82, 1428) 481
Med Tx/ Mod	2	123 (31, 554) 185	326 (83, 1449) 488
Med Tx/ Mod	3	138 (36, 606) 205	370 (96, 1610) 549
Med Tx/ Mod	4	164 (42, 723) 244	421 (108, 1824) 626
High Tx/ Mild	1	27 (8, 93) 36	76 (23, 262) 101
High Tx/ Mild	2	27 (8, 93) 36	76 (23, 262) 102
High Tx/ Mild	3	29 (9, 98) 39	82 (25, 281) 109
High Tx/ Mild	4	34 (10, 114) 45	91 (27, 309) 120
High Tx/ Mod	1	323 (90, 1000) 428	888 (244, 3889) 1319
High Tx/ Mod	2	324 (90, 1000) 430	893 (246, 3903) 1327
High Tx/ Mod	3	345 (96, 1000) 449	959 (266, 4248) 1431
High Tx/ Mod	4	403 (111, 1000) 499	1065 (292, 4937) 1619
High Tx/ Sev	1	1000 (1000, 1000) 999	25603 (4305, 27600) 19615
High Tx/ Sev	2	1000 (1000, 1000) 999	25858 (4341, 27600) 19664
High Tx/ Sev	3	1000 (1000, 1000) 999	27600 (4753, 27600) 20207
High Tx/ Sev	4	1000 (1000, 1000) 1000	27600 (5557, 27600) 21109

Table S6: Peak hospital utilisation is reported separately for ICUs and general wards; the maximum capacities are 1,000 and 27,600 beds, respectively. **Key message:** ICU capacity is only exceeded in high-severity scenarios, while general ward capacity is only exceeded in the high-transmission high-severity (worst case) scenario. Liberal antiviral use in the community ("Rx All/PEP Eld, HR" and "Rx All" strategies) can reduce the hospital burden.

Scenario	Strategy	ICU Utilisation	Ward Utilisation
Low Tx/ Mild	1	158 (19, 692) 233	420 (50, 1828) 618
Low Tx/ Mild	2	163 (20, 702) 238	432 (54, 1854) 631
Low Tx/ Mild	3	199 (40, 794) 282	533 (110, 2121) 756
Low Tx/ Mild	4	242 (55, 941) 339	619 (143, 2386) 864
Low Tx/ Mod	1	1709 (158, 9465) 2842	4485 (420, 24318) 7365
Low Tx/ Mod	2	1765 (173, 9664) 2927	4621 (460, 24805) 7571
Low Tx/ Mod	3	2272 (423, 11118) 3583	6005 (1143, 28878) 9383
Low Tx/ Mod	4	2836 (597, 13550) 4402	7108 (1521, 33161) 10936
Low Tx/ Sev	1	12993 (145, 123102) 28788	32951 (362, 404972) 91186
Low Tx/ Sev	2	14725 (145, 128318) 31208	37251 (362, 443462) 101102
Low Tx/ Sev	3	32279 (1333, 156164) 52708	82310 (3432, 845478) 197733
Low Tx/ Sev	4	48493 (4679, 167957) 66408	116724 (11497, 1176200) 283869
Med Tx/ Mod	1	5158 (1508, 19953) 7328	13427 (3967, 51165) 18948
Med Tx/ Mod	2	5206 (1521, 20128) 7389	13536 (4000, 51533) 19086
Med Tx/ Mod	3	5592 (1630, 21618) 7922	14755 (4332, 56089) 20727
Med Tx/ Mod	4	6507 (1894, 25470) 9270	16320 (4789, 62711) 23064
High Tx/ Mild	1	663 (216, 2067) 850	1756 (571, 5453) 2248
High Tx/ Mild	2	665 (216, 2073) 853	1761 (572, 5470) 2253
High Tx/ Mild	3	697 (226, 2170) 894	1866 (606, 5795) 2390
High Tx/ Mild	4	800 (259, 2491) 1025	2037 (661, 6323) 2606
High Tx/ Mod	1	7865 (2321, 27141) 10748	20521 (6104, 80036) 29133
High Tx/ Mod	2	7906 (2329, 27182) 10779	20557 (6112, 80224) 29218
High Tx/ Mod	3	8277 (2440, 27883) 11210	21851 (6487, 85782) 31045
High Tx/ Mod	4	9558 (2807, 30185) 12655	23886 (7095, 97601) 34481
High Tx/ Sev	1	53644 (30919, 93677) 56719	603711 (104847, 1481173) 669137
High Tx/ Sev	2	53499 (30938, 92672) 56471	605288 (105224, 1471503) 668297
High Tx/ Sev	3	53229 (31314, 87888) 55435	630356 (112720, 1449222) 678535
High Tx/ Sev	4	54322 (32959, 87684) 56466	688154 (128880, 1468331) 714117

Table S7: Cumulative hospital utilisation is reported separately for ICUs and general wards. **Key message:** Liberal antiviral use in the community (“Rx All/PEP Eld, HR” and “Rx All” strategies) can reduce the hospital burden, but in the high-severity scenarios these reductions are only minor when compared to the scale of the epidemic.

Scenario	Strategy	High-Risk Deaths	Total Deaths
Low Tx/ Mild	1	4 (0, 17) 6	8 (1, 34) 11
Low Tx/ Mild	2	4 (1, 18) 6	8 (1, 34) 12
Low Tx/ Mild	3	5 (1, 19) 7	10 (2, 38) 14
Low Tx/ Mild	4	7 (2, 27) 10	12 (3, 47) 17
Low Tx/ Mod	1	45 (4, 257) 76	84 (8, 467) 140
Low Tx/ Mod	2	46 (5, 265) 79	87 (9, 478) 145
Low Tx/ Mod	3	58 (11, 294) 93	112 (21, 546) 176
Low Tx/ Mod	4	83 (17, 415) 132	143 (30, 685) 222
Low Tx/ Sev	1	378 (4, 4538) 1069	658 (7, 7719) 1794
Low Tx/ Sev	2	427 (4, 5045) 1200	743 (7, 8467) 1997
Low Tx/ Sev	3	917 (39, 8236) 2077	1618 (69, 14373) 3609
Low Tx/ Sev	4	1591 (159, 12995) 3413	2504 (250, 20459) 5349
Med Tx/ Mod	1	134 (38, 547) 196	253 (74, 986) 361
Med Tx/ Mod	2	136 (39, 554) 198	255 (74, 994) 364
Med Tx/ Mod	3	141 (40, 568) 205	272 (79, 1061) 388
Med Tx/ Mod	4	191 (54, 776) 278	326 (94, 1283) 467
High Tx/ Mild	1	16 (5, 52) 21	32 (10, 101) 41
High Tx/ Mild	2	17 (5, 52) 21	32 (10, 101) 41
High Tx/ Mild	3	17 (5, 52) 21	34 (11, 105) 43
High Tx/ Mild	4	22 (7, 70) 29	40 (13, 124) 51
High Tx/ Mod	1	205 (59, 828) 298	385 (113, 1488) 548
High Tx/ Mod	2	206 (59, 832) 299	387 (113, 1496) 550
High Tx/ Mod	3	209 (60, 840) 302	404 (118, 1558) 572
High Tx/ Mod	4	280 (80, 1127) 405	478 (140, 1863) 680
High Tx/ Sev	1	6671 (1110, 62967) 15400	11077 (1952, 87849) 23180
High Tx/ Sev	2	6717 (1118, 63409) 15499	11112 (1960, 88400) 23290
High Tx/ Sev	3	6302 (1128, 57863) 14430	10877 (2040, 84924) 22795
High Tx/ Sev	4	7982 (1516, 60049) 16803	12603 (2447, 88091) 25317

Table S8: Deaths are reported for the High-Risk stratum and for the entire population. **Key message:** In all scenarios, liberal antiviral use in the community ("Rx All/PEP Eld, HR", "Rx All" and "Rx At-Risk, Hosp" strategies) can prevent more deaths than is achieved by providing treatment solely to hospitalised cases ("Rx Hosp" strategy).

Scenario	Strategy	Presentation	Hospitalisation
Low Tx/ Mild	1	0.845 (0.273, 0.959) 0.763	0.734 (0.237, 0.833) 0.663
Low Tx/ Mild	2	0.858 (0.295, 0.965) 0.779	0.748 (0.258, 0.841) 0.680
Low Tx/ Mild	3	0.956 (0.714, 0.985) 0.921	0.875 (0.654, 0.902) 0.843
Low Tx/ Mild	4	0.998 (0.916, 1.000) 0.986	0.998 (0.916, 0.999) 0.986
Low Tx/ Mod	1	0.810 (0.167, 0.950) 0.715	0.700 (0.144, 0.822) 0.618
Low Tx/ Mod	2	0.827 (0.190, 0.957) 0.733	0.718 (0.165, 0.831) 0.637
Low Tx/ Mod	3	0.939 (0.599, 0.980) 0.891	0.854 (0.544, 0.893) 0.811
Low Tx/ Mod	4	0.987 (0.835, 0.997) 0.965	0.987 (0.835, 0.997) 0.965
Low Tx/ Sev	1	0.376 (0.001, 0.891) 0.394	0.320 (0.001, 0.763) 0.337
Low Tx/ Sev	2	0.437 (0.001, 0.902) 0.419	0.375 (0.001, 0.777) 0.360
Low Tx/ Sev	3	0.771 (0.020, 0.945) 0.641	0.693 (0.018, 0.851) 0.577
Low Tx/ Sev	4	0.879 (0.104, 0.976) 0.766	0.879 (0.104, 0.976) 0.766
Med Tx/ Mod	1	0.955 (0.855, 0.989) 0.942	0.827 (0.738, 0.858) 0.816
Med Tx/ Mod	2	0.959 (0.862, 0.991) 0.946	0.832 (0.748, 0.862) 0.822
Med Tx/ Mod	3	0.980 (0.952, 0.993) 0.977	0.892 (0.864, 0.906) 0.890
Med Tx/ Mod	4	0.996 (0.987, 0.999) 0.995	0.996 (0.987, 0.999) 0.995
High Tx/ Mild	1	0.989 (0.951, 0.998) 0.983	0.860 (0.828, 0.869) 0.856
High Tx/ Mild	2	0.990 (0.953, 0.998) 0.984	0.863 (0.831, 0.870) 0.858
High Tx/ Mild	3	0.994 (0.984, 0.998) 0.993	0.909 (0.900, 0.913) 0.908
High Tx/ Mild	4	0.999 (0.999, 1.000) 0.999	0.999 (0.995, 0.999) 0.998
High Tx/ Mod	1	0.986 (0.942, 0.997) 0.980	0.854 (0.816, 0.866) 0.849
High Tx/ Mod	2	0.987 (0.943, 0.997) 0.981	0.856 (0.819, 0.867) 0.851
High Tx/ Mod	3	0.992 (0.978, 0.997) 0.990	0.902 (0.887, 0.910) 0.901
High Tx/ Mod	4	0.998 (0.993, 0.999) 0.997	0.997 (0.992, 0.999) 0.996
High Tx/ Sev	1	0.962 (0.854, 0.990) 0.947	0.825 (0.730, 0.852) 0.812
High Tx/ Sev	2	0.963 (0.855, 0.990) 0.947	0.827 (0.735, 0.854) 0.815
High Tx/ Sev	3	0.971 (0.904, 0.991) 0.962	0.872 (0.811, 0.895) 0.865
High Tx/ Sev	4	0.982 (0.933, 0.995) 0.975	0.981 (0.933, 0.994) 0.975

Table S9: The relative risks of presenting (at either an outpatient or inpatient setting) and of requiring hospitalisation (regardless of actual bed capacity) are shown below. These relative risks are calculated with respect to identical pandemic scenarios in the absence of antiviral interventions for both the Initial Action and Targeted Action phases. **Key message:** Antiviral interventions produce minor reductions in the relative risk of presentation (except when the intervention can mitigate the epidemic), but can significantly reduce the relative risk of hospitalisation in even the most severe scenarios through the provision of early treatment (in the community) to cases that would otherwise require hospitalisation.

Scenario	Strategy	ICU Admission	Death
Low Tx/ Mild	1	0.527 (0.171, 0.599) 0.477	0.352 (0.115, 0.400) 0.318
Low Tx/ Mild	2	0.538 (0.186, 0.605) 0.489	0.359 (0.125, 0.404) 0.327
Low Tx/ Mild	3	0.623 (0.466, 0.642) 0.601	0.413 (0.310, 0.426) 0.398
Low Tx/ Mild	4	0.741 (0.681, 0.743) 0.732	0.505 (0.465, 0.506) 0.499
Low Tx/ Mod	1	0.498 (0.103, 0.586) 0.441	0.330 (0.069, 0.389) 0.292
Low Tx/ Mod	2	0.512 (0.118, 0.593) 0.454	0.339 (0.078, 0.394) 0.302
Low Tx/ Mod	3	0.602 (0.383, 0.633) 0.573	0.397 (0.253, 0.418) 0.378
Low Tx/ Mod	4	0.727 (0.615, 0.739) 0.712	0.491 (0.417, 0.502) 0.482
Low Tx/ Sev	1	0.223 (0.001, 0.538) 0.236	0.146 (0.000, 0.353) 0.155
Low Tx/ Sev	2	0.263 (0.001, 0.549) 0.253	0.172 (0.000, 0.361) 0.167
Low Tx/ Sev	3	0.480 (0.012, 0.593) 0.400	0.312 (0.008, 0.388) 0.261
Low Tx/ Sev	4	0.637 (0.075, 0.711) 0.556	0.426 (0.050, 0.477) 0.372
Med Tx/ Mod	1	0.589 (0.525, 0.614) 0.581	0.390 (0.348, 0.408) 0.385
Med Tx/ Mod	2	0.593 (0.533, 0.617) 0.586	0.393 (0.353, 0.410) 0.389
Med Tx/ Mod	3	0.630 (0.607, 0.643) 0.628	0.415 (0.398, 0.425) 0.414
Med Tx/ Mod	4	0.735 (0.722, 0.740) 0.733	0.498 (0.485, 0.503) 0.496
High Tx/ Mild	1	0.619 (0.595, 0.625) 0.615	0.413 (0.397, 0.418) 0.411
High Tx/ Mild	2	0.621 (0.598, 0.626) 0.617	0.414 (0.399, 0.418) 0.412
High Tx/ Mild	3	0.647 (0.641, 0.651) 0.647	0.429 (0.425, 0.432) 0.429
High Tx/ Mild	4	0.742 (0.738, 0.743) 0.741	0.505 (0.502, 0.506) 0.505
High Tx/ Mod	1	0.608 (0.580, 0.621) 0.605	0.403 (0.383, 0.413) 0.401
High Tx/ Mod	2	0.610 (0.582, 0.622) 0.607	0.404 (0.385, 0.414) 0.403
High Tx/ Mod	3	0.637 (0.622, 0.646) 0.636	0.420 (0.407, 0.428) 0.419
High Tx/ Mod	4	0.735 (0.725, 0.740) 0.734	0.498 (0.488, 0.503) 0.497
High Tx/ Sev	1	0.591 (0.512, 0.766) 0.616	0.389 (0.335, 0.724) 0.457
High Tx/ Sev	2	0.593 (0.515, 0.769) 0.618	0.390 (0.337, 0.727) 0.459
High Tx/ Sev	3	0.615 (0.566, 0.779) 0.636	0.402 (0.367, 0.720) 0.453
High Tx/ Sev	4	0.719 (0.685, 0.857) 0.733	0.482 (0.457, 0.759) 0.517

Table S10: The relative risks of requiring ICU admission (regardless of actual ICU capacity) and death are shown below. These relative risks are calculated with respect to identical pandemic scenarios in the absence of antiviral interventions for both the Initial Action and Targeted Action phases. **Key message:** The relative risks of ICU admission and deaths are substantially reduced when treatment is only provided to hospitalized cases ("Rx Hosp" strategy). Additional provision of treatment to community presentations reduce these risks even further — compare the relative risks in the worst case scenario (high-transmission high-severity) for the liberal strategies ("Rx All/PEP Eld HR" and "Rx All") to the "Rx Hosp" strategy.

S2 Model equations

The transmission model used in this paper involves one major modification to the contact model first introduced in [1] and further developed in [2, 3]. In this study, the population was stratified into five distinct risk groups (young children, elderly, high-risk, health care workers, and the general adult population), to allow for differential risks of severe outcomes, differential benefits conferred by antiviral treatment, and targeted treatment and prophylaxis strategies. We assumed these groups mixed homogeneously. The model structure is shown in Figure S1.

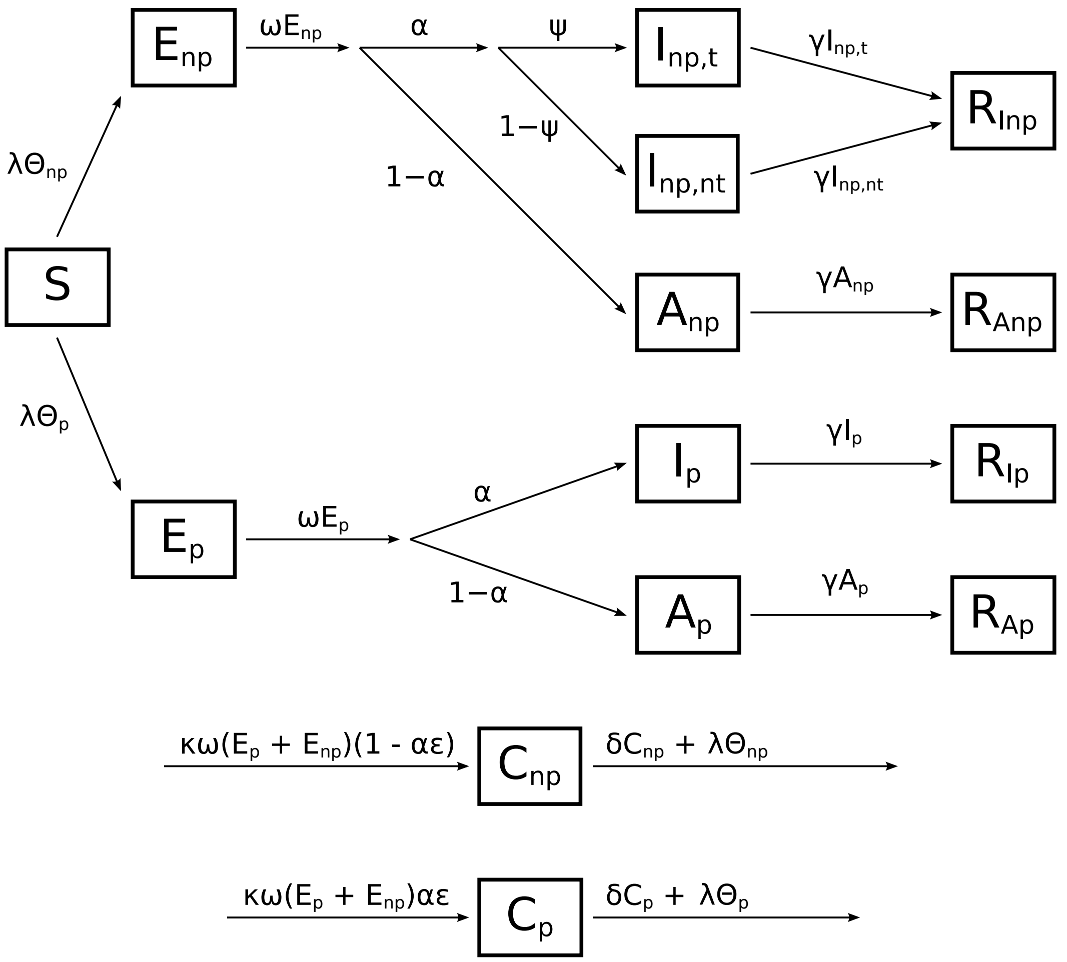

Figure S1: The flow between the state variables in the model where ψ and ϵ are functions of time, as introduced in [3]. The contact classes C_{np} and C_p are labels for tracking contact status and are orthogonal to the SEIR states; see [1] for further details.

The proportion of all infected cases that present (α) is the sum of the severe cases (all of which present) and the proportion (α_M) of the remaining (i.e., mild) cases that present:

$$\alpha = \eta + \alpha_M(1-\eta) \tag{S1}$$

We have assumed that α_M is dependent on the severity of the epidemic (η), as first defined in [3] and illustrated in Figure S2.

In recognition that not all contacts of an infectious individual can be identified and provided with post-exposure prophylaxis, the parameter σ defines the proportion of contacts that are potentially identifiable. Accordingly, the proportion of all contacts that receive prophylaxis (ϵ) cannot exceed σ.

Finally, the fraction of presenting cases that receive treatment (ψ) and the fraction of contacts that receive prophylaxis (ϵ) are functions of time, since they are both affected by the logistical constraints introduced in this model and do not remain constant throughout an epidemic.

The original SEIR model [2] introduced Θ_p and Θ_{np}, which define the proportion of susceptible contacts in the population:

$$\Theta_p = \frac{e_s C_p}{C_P + C_{np}} \times \frac{S}{N} \tag{S2}$$

$$\Theta_{np} = \frac{C_{np}}{C_P + C_{np}} \times \frac{S}{N} \tag{S3}$$

The force of infection (λ) arises from the five infectious classes just as in the original SEIR model [2], given the number of infections per unit time made by an infectious individual (β):

$$\beta = R_0 \times \gamma \tag{S4}$$

$$\lambda_p = \beta e_i(I_p + A_p) \tag{S5}$$

$$\lambda_{np,nt} = \beta(I_{np,nt} + A_{np}) \tag{S6}$$

$$\lambda_{np,t} = \beta e_t I_{np,t} \tag{S7}$$

$$\lambda = \lambda_p + \lambda_{np,nt} + \lambda_{np,t} \tag{S8}$$

We have previously extended this model to include vaccination [3], but for the purposes of this study we assumed that no vaccine would be available.

S3 Latin hypercube sampling

Note that the combination of α_{rel} and the most conservative value of e_s provides an upper bound matched to estimates of prevention of clinical symptoms and a lower bound consistent with greatest efficacy in household settings (Jefferson et al., *Cochrane Database Syst Rev*, 4:CD008965, 2014).

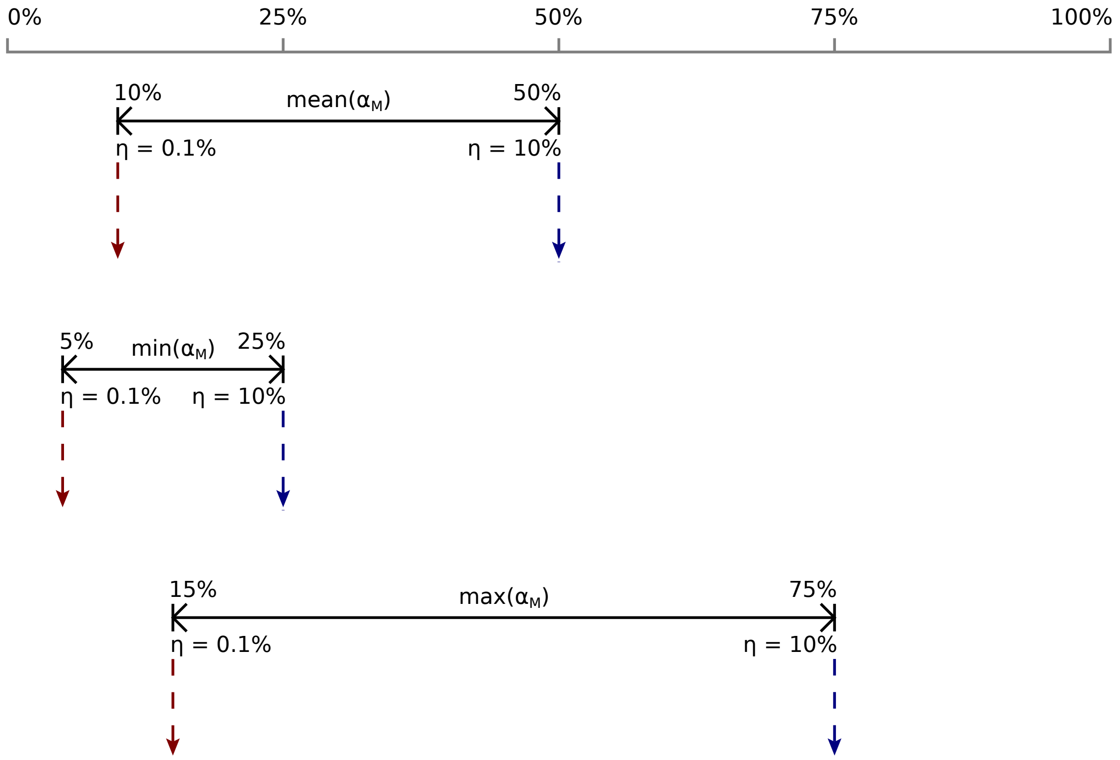

(a) The mean, minimum and maximum values for α_m are linear functions of η ($\eta \in [0.1\%, 10\%]$).

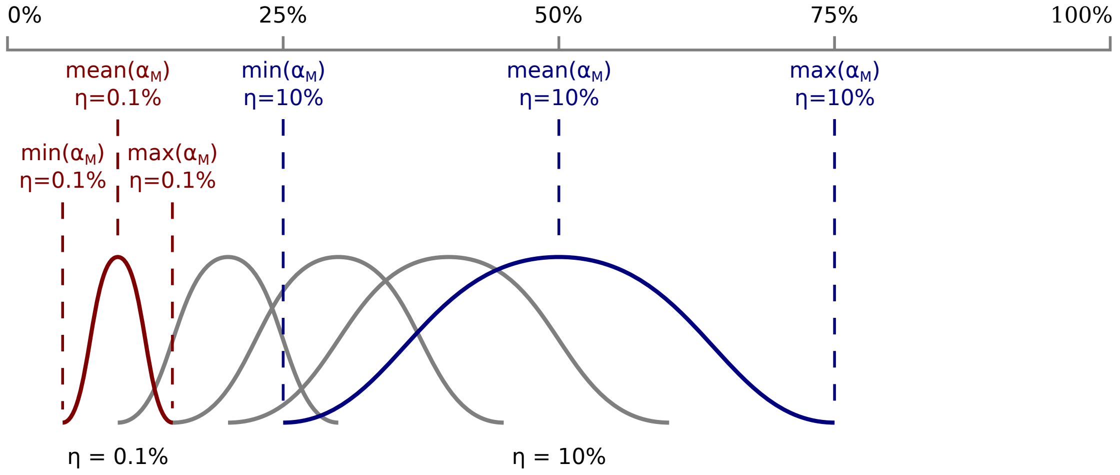

(b) The probability distribution for α_m when $\eta = 0.1\%$, 2.575%, 5.05%, 7.525% and 10%; the distributions for $\eta = 0.1\%$ and $\eta = 10\%$ are shown in red and blue. This distribution is given by: $\min(\alpha_m) + [\max(\alpha_m) - \min(\alpha_m)] \times \text{Beta}(\mu = 0.5, V = 0.2)$.

Figure S2: The probability distribution for mild presentations (α_M).

	$A + B \times \mathrm{Beta}(\mu, V)$				
	A	B	μ	V	Meaning
ω	180	550	0.3364	0.1636	Inverse latent period (years^{-1})
γ	146	219	0.4444	0.2283	Inverse infectious period (years^{-1})
δ	121.6667	60.8333	0.4	0.1973	Inverse contact period (years^{-1})
e_t	0.5	0.4	0.5	0.25	Relative infectiousness of treated cases
e_i	0.5	0.4	0.5	0.25	Relative infectiousness of breakthrough cases
e_s	0.4	0.6	0.7	0.1	Relative susceptibility of those given PEP

	$\mathcal{U}(a, b)$		
	a	b	
O	1×10^6	20×10^6	Antiviral stockpile size
σ	0.1	0.5	Fraction of contacts that can receive PEP
μ	0.5	1.0	Compliance at out-patient facilities

	$\exp_{10}[\mathcal{U}(a, b)]$		
	10^a	10^b	
D_c	10^3	10^5	Laboratory test capacity (per day)

	Value	
N	20×10^6	Population size
κ	20	Average number of contacts (per day)
χ	1	Relative infectiousness of sub-clinical cases
α_{rel}	0.5	Propensity of breakthrough cases to display symptoms and present
e_{dp}	0.7	Effectiveness of delayed PEP
e_{dt}	0.7	Effectiveness of delayed treatment

Table S11: Model parameter distributions used in all pandemic scenarios. Each parameter is associated with a beta distribution (top), a uniform distribution (second), a log-uniform distribution (third), or a single value (bottom).

References

[1] James M. McCaw and Jodie McVernon. Prophylaxis or treatment? optimal use of an antiviral stockpile during an influenza pandemic. *Mathematical Biosciences*, 209 (2):336–360, Oct 2007. ISSN 0025-5564, doi:10.1016/j.mbs.2007.02.003.

[2] Jodie McVernon, James M. McCaw, and Terence M. Nolan. Modelling strategic use of the national antiviral stockpile during the CONTAIN and SUSTAIN phases of an Australian pandemic influenza response. *Australian and New Zealand Journal of Public Health*, 34(2):113–119, Apr 2010. ISSN 1753-6405, doi:10.1111/j.1753-6405.2010.00493.x.

[3] Robert Moss, James M. McCaw, and Jodie McVernon. Diagnosis and antiviral

intervention strategies for mitigating an influenza epidemic. *PLOS ONE*, 6(2):e14505, Feb 2011. ISSN 1932-6203, doi:10.1371/journal.pone.0014505.
